# Urea supplementation improves mRNA in vitro transcription by decreasing both shorter and longer RNA byproducts

**DOI:** 10.1080/15476286.2024.2321764

**Published:** 2024-02-27

**Authors:** Combes Francis, Pettersson Frida J, Bui Thanh-Huong, Molska Alicja, Komissarov Artem, Parot Jérémie, Borgos Sven Even

**Affiliations:** Department of Biotechnology and Nanomedicine, SINTEF, Trondheim, Norway

**Keywords:** In vitro transcription, urea, VSW3, RNA polymerase, transcript impurities, mRNA byproducts

## Abstract

The current letter to the editor describes the presence of RNA byproducts in small-scale in vitro transcription (IVT) reactions as evaluated by capillary gel electrophoresis, asymmetric flow field flow fractionation, immunoblotting, cell-free translation assays, and in IFN reporter cells. We compare standard T7 RNA polymerase (RNAP) based IVT reactions to two recently described protocols employing either urea supplementation or using the VSW3 RNAP. Our results indicate that urea supplementation yields considerably less RNA byproducts and positively affects the overall number of full-length transcripts. In contrast, VSW3 IVT reactions demonstrated a low yield and generated a higher fraction of truncated transcripts. Lastly, both urea mRNA and VSW3 mRNA elicited considerably less IFN responses after transfection in mouse macrophages.

## Introduction

The bacteriophage T7 RNA polymerase (RNAP) is the most widely used enzyme to perform run-off in vitro transcription (IVT). Optimization of e.g. Mg^2+^ concentration, IVT incubation time, RNAP:template ratio, and nucleotide concentration (especially UTP) to suppress the number of aberrant transcripts has been extensively explored [1,2]. Yet, considerable downstream purification is needed to remove unwanted byproducts. Examples of such byproducts include abortive and truncated transcripts, 3’-extended transcripts, and dsRNA (Table 1) [1,2,4]. In therapeutic contexts, these byproducts are known to elicit unwanted immune reactions, which lead to translational suppression and adverse effects. Nevertheless, state-of-the-art mRNA production, which includes 5’ dephosphorylation of uncapped transcripts [2,7], dsRNA removal by cellulose [8], and/or chromatographic purification [9], still results in immunogenic mRNA able to trigger MDA5-mediated immune response [10]. Although the exact source of this response is not yet fully elucidated, it is likely that residual dsRNA species are still present in highly purified mRNA. Many attempts are made to both identify this contaminant and to find RNAPs that result in lower production of dsRNA [2,4,5,11]. Examples of the latter include rationally engineered T7 RNAP [12], thermostable T7 RNAP [5], and the psychrophilic phage VSW3 RNAP [13].

**Table 1.** Origin of IVT mRNA byproducts.

Byproduct	Cause
Abortive transcripts	Unstable RNAP conformation during transcription initiation; unsuccessful displacement of the nascent mRNA from the DNA template; and downstream transcription bubble collapse [3].
Truncated transcripts	Incomplete run-off transcripts at the end of the IVT reaction and transcription abortion due to e.g. RNAP terminator sequences and unstable T7 RNAP conformation at the end of the DNA template [4].
Degraded transcripts	Enzyme-independent hydrolysis or RNase contamination.
3’ transcript extensions	Template-independent elongation (e.g. in excess of NTPs), RNA template-dependent elongation in *cis* (i.e. folding back on the same RNA molecule) or to a lower degree in *trans* (annealing to a 2^nd^ RNA molecule [5]), RNAP switching from the sense DNA to the antisense DNA at the 3’ terminus [5,6], accumulation of run-off transcripts [4], and incompletely digested plasmid template DNA.
dsRNA formation	Transcript extensions (especially RNA looping back on itself in *cis*, [4]), promoter-independent transcription from a DNA end [2], promoter-independent transcription primed by short transcripts [4], and excessive Mg^2+^ [2].

The VSW3 RNAP was recently isolated and characterized [13]. This enzyme is reported to efficiently produce transcripts at reaction temperatures between 4°C and 25°C, with a maximal yield comparable to that of the T7 RNAP. Importantly, VSW3 RNAP appears to be less sensitive to transcription terminators, 3’ extensions, and dsRNA generation. It is, however, not clear to what extent VSW3 RNAP can also incorporate N1-methylpseudouridine (m1Ψ) into synthetic RNA, a prerequisite for *in vivo* application of the IVT mRNA. Moreover, whereas T7 RNAP IVT is known to generate less dsRNA and 3’ extensions at temperatures > 37°C [5], elevated temperature also increases the rate of hydrolytic cleavage and hence advocates for lower temperature IVT reactions such as the 25°C VSW3 RNAP-based IVT reaction. Similarly, post-IVT chromatography at high temperature or considerable shear force may affect overall mRNA integrity resulting in lower protein expression [14].

Another strategy to prevent aberrant transcript formation during IVT is the addition of denaturing agents in the IVT reaction. For example, formamide or urea supplementation have been demonstrated to result in 60–70% lower dsRNA generation without substantially affecting mRNA yield or nucleotide misincorporation rate [14]. Such dsRNA content analysis is typically performed through immunoblotting assays using the J2 antibody [15]. This antibody, however, cannot detect dsRNA shorter than 43 bp, and it is unclear to what extent the J2 mAb binds ssRNA secondary structures [14–16]. Analytical methods to detect dsRNA include HPLC, mass spectrometry (MS), and asymmetric field flow fractionation (AF4) [11,17,18].

In the current investigation, we demonstrate that the recently reported VSW3 polymerase struggles when using m1Ψ in the IVT and is therefore, in our hands, not a viable option to produce mRNA for *in vivo* use. However, the addition of urea to a T7 RNAP IVT reaction decreased transcript impurities while maintaining high mRNA yield.

## Results

### IVT YIELD and CRUDE QUALITY

**Figure 1.**
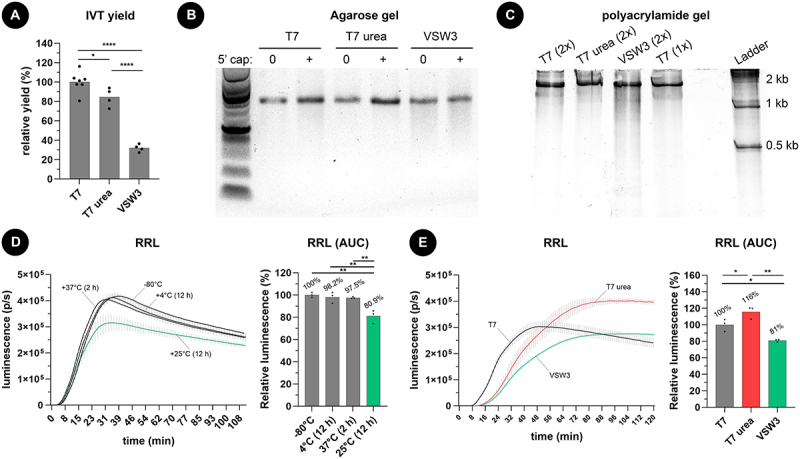
A) as measured by spectrophotometry, one molar urea supplementation to T7 RNAP-based IVT reactions (2 hours at 37°C) has a minor impact on mRNA yield. In contrast, VSW3 RNAP-based IVT (16 hours at 25°C) results in significantly less mRNA. The dots represent independent IVT reactions, and all reactions employed m1Ψ. B) agarose gel electrophoresis reveals a smear of shorter mRNA transcripts in VSW3 IVT, both before and after a vaccinia capping reaction. C) smear analysis on 5% polyacrylamide gel indicates that an additional silica purification (2×) does not substantially remove shorter transcripts. PAGE further confirms the presence of many shorter transcripts in VSW3 mRNA. D) commercially acquired CleanCap® FLuc mRNA (TriLink BioTechnologies) was subjected to heat at the indicated temperature and time. 500 ng of this mRNA was then translated in a rabbit reticulocyte lysate assay (RRL) in the presence of substrate D-luciferin (3 technical replicates, bars indicate SD). E) the protein production of 500 ng capped and silica-purified fluc mRNA was used in an RRL reaction as described in figure 1D. Analysis of the AUC of these graphs indicates a higher number of full-length transcripts in the urea-supplemented T7 mRNA and significantly less functional fluc-encoding transcripts in the VSW3 IVT mRNA (3 technical replicates, bars indicate SD).

Spectrophotometer measurements of silica-purified transcripts (Figure 1A) indicated that 1 M urea supplementation decreases the overall T7 IVT yield from 100% (the yield of a standard T7 IVT) to 84.4%. More striking is that the overall IVT yield of the VSW3 reactions dropped to 31.9%. We additionally noticed a slight smear below the VSW3 mRNA bands on agarose gel (Figure 1B). This smear of shorter mRNA transcripts (reaching below 500 nt) in the VSW3 sample was further confirmed on a native polyacrylamide gel. In contrast, urea T7 IVT demonstrated a decrease in truncated transcripts compared to control T7 IVT (Figure 1C). To further investigate the cause of the smear, we performed VSW3 and T7 IVT reactions in the presence or absence of RNase inhibitor. We also included a control where purified mRNA was incubated in parallel with the VSW3 reaction (i.e. 16 hours at 25°C). Subsequent PAGE indicates that the smear can largely be attributed to the long incubation times and that RNAse inhibitor supplementation does not remove the smear from VSW3 IVT mRNA (Supplement Figure S1A). Interestingly, when we prolong the T7 IVT reaction to 16 hours, we also see a higher fraction of shorter transcripts appearing, further supporting our assumption that these contaminants mainly appear after long incubation (Supplement Figure S1B). We excluded the presence of mutations in the VSW3 promoter sequence by Sanger Sequencing (Supplement Figure S1C) and discovered that using uridine triphosphate rather than m1Ψ triphosphate results in a 2.3-fold higher yield of VSW3 IVT transcripts (corresponding to a relative VSW3 IVT yield of 73.4%, Supplement Figure S1D).

To investigate the effect of prolonged heating on mRNA integrity, we compared the protein output of mRNA encoding firefly luciferase (fluc) fresh from storage (−80°C) with mRNA incubated at 4–37°C for 2–12 hours (Figure 1D). Incubating mRNA for 12 hours at 25°C prior to cell-free translation resulted in a 19.1% loss of full-length, fully translatable mRNA while only 2.5% loss was detected after incubating mRNA at 37°C for 2 hours. Performing the cell-free translation assay on mRNA obtained after T7, urea T7, and VSW3 IVT reactions further showed that urea T7 mRNA produced the highest amount of protein (116%) compared to T7 mRNA (100%) and VSW3 mRNA (81%) (Figure 1E). Overall, these results demonstrate that, for a fixed mass of 500 ng mRNA, urea T7 mRNA contains a higher fraction of translatable mRNAs whereas VSW3 mRNA contains fewer translatable mRNA strands. The latter can be correlated with the long (>12 hours) incubation time needed to perform the VSW3 IVT reaction.

### CGE, AF4 BLOT, and IFN response

**Figure 2.**
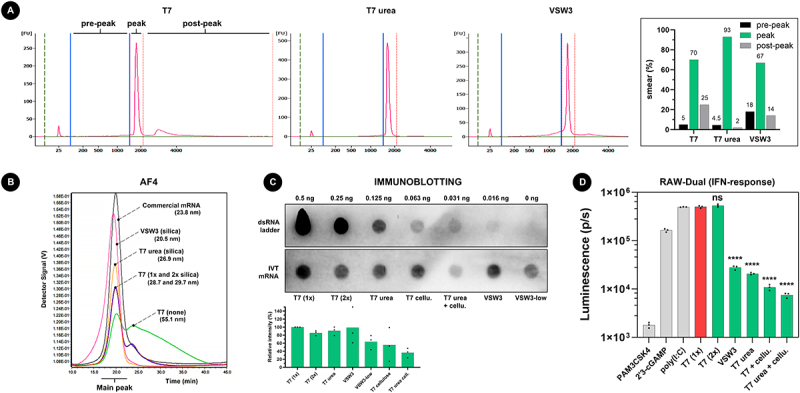
A) addition of 1 M urea to the IVT reaction decreases both shorter and longer transcript impurities. 200 ng of IVT mRNA was loaded per lane on an agilent bioanalyzer 2100 system in non-denaturing conditions. The indicated pre-peak, peak, and post-peak regions on the electropherogram were used during smear analysis (right graph). B) AF4 analysis of about 500 ng mRNA per sample confirms that VSW3 mRNA is mainly contaminated with transcripts of low MW. In contrast, urea T7 mRNA is cleared from both low and high MW transcripts. The measured radius of gyration of each sample is listed between parentheses. C) Representative immunoblot using the J2 antibody. 5 µg mRNA was analysed per sample and compared to the intensity of a dsRNA ladder dilution. The bottom graph depicts the average relative intensities of 3 independent dot blot assays (one-way ANOVA *p* = 0.0556). D) RAW-DualTM cells were transfected with controls (grey bars) or IVT mRNA samples (green bars). The asterisks indicate statistically lower luminescence compared to the reference T7 (1×) sample (red bar). Additional pairwise comparisons (excluding the controls) were performed but did not find significant differences after correction for multiple comparisons.

To investigate the presence of longer transcripts and to also have a semi-quantitative notion of the IVT mRNA, we first performed capillary gel electrophoresis (CGE) using the Agilent Bioanalyzer 2100 system (Figure 2A). As expected, VSW3 mRNA demonstrated the highest smear percentage of shorter ‘pre-peak’ mRNA (18% of total RNA content) but also showed a decrease in the percentage of longer ‘post-peak’ mRNA (14%) compared to standard T7 mRNA (25%). Whereas urea supplementation only modestly decreases the percentage of shorter RNAs (4.5%) compared to standard T7 IVT (5%), a considerably lower percentage of longer RNA can be seen (2%). Asymmetric field flow fractionation (AF4) further confirmed these data by first demonstrating a distinct reference peak of oligo-dT-purified commercial CleanCap® FLuc mRNA (Trilink) and an equally well-defined peak for T7 urea mRNA (Figure 2B). In contrast, both unpurified and silica purified T7 mRNA showed a clear second peak which indicates the presence of high molecular weight RNA. Like T7 urea mRNA, this second peak was virtually absent in VSW3 mRNA but a more spread-out front of the peak reveals the presence of RNA byproducts with lower molecular weight.

Compared to regular T7 transcripts that were purified once on a silica column (100% relative intensity, corresponding to 21.8 pg dsRNA per µg input mRNA), a second silica purification does not majorly decrease the apparent dsRNA content after immunoblotting (85.3%), nor does urea supplementation during IVT (90.8%) or when VSW3 RNAP was employed (98.8%). Interestingly, a more pronounced decrease in dsRNA is suggested when using 10-fold less pyrophosphatase during IVT (i.e. 0.002 units, VSW3-low, 63.7%). Additional dsRNA removal is seen when cellulose purification is used, both after a regular T7 IVT reaction (55.5%) and after urea T7 IVT (36.3%). Transfection of these mRNA samples into the RAW-Dual^TM^ murine macrophage reporter cell line demonstrated that mRNA produced by urea supplementation or by using the VSW3 polymerase leads to lower interferon responses (Figure 2D) and that purifying the mRNA with cellulose further reduced the signal. In contrast, none of the mRNA samples induced NF-kB responses in this reporter cell line (Supplement Figure S1E).

## Discussion

Producing mRNA transcripts for *in vivo* applications requires the use of modified uridine (typically m1Ψ) and the removal of immunogenic transcript impurities [2,17]. mRNA purification procedures which selectively remove dsRNA by cellulose affinity or chromatographic separation are therefore warranted. These techniques, however, are rather impractical to carry out or require extensive resources and expertise [5]. This led several research groups to investigate the use of alternative RNAPs and reaction conditions to minimize RNA byproduct formation during IVT. The current report follows this trend by exploring two recently (2022) published strategies to produce m1Ψ-modified mRNA: (a) employing the VSW3 RNAP in IVT reactions [13] and (b) supplementing 1 M urea to T7 IVT reactions [14].

Using two different batches of VSW3 RNAP, our findings demonstrate that VSW3 IVT exhibits a low overall yield with a relatively high fraction of short transcripts which subsequently decreased the protein production capacity. The short transcripts mainly appear after prolonged exposure to aqueous environments and are not associated with RNase-mediated degradation. Additional immunoblotting and transfections into reporter cells further suggested that VSW3 IVT mRNA also contained less immunogenic dsRNA. Using 10-fold less pyrophosphatase is known to inhibit the IVT reaction [19] and it further reduced the immunoblotting signal. Similarly, VSW3 mRNA demonstrated a lower fraction of long (CGE) and/or high molecular weight transcripts (AF4). The experimental results using VSW3 mRNA are consistent with an overall low polymerization speed of the VSW3 RNAP at 25°C, particularly when using m1Ψ during IVT. This conclusion explains the long incubation time needed, the low yield obtained, the higher number of truncated transcripts detected, and the lower amount of dsRNA transcripts seen (which are primarily attributed to loop-back transcription [4].

As an alternative, 1 M urea supplementation to the standard T7 IVT reaction (employing m1Ψ) appeared to decrease the number of short transcripts and only minimally affected the overall mRNA yield. CGE and AF4 further indicated that urea supplementation also resulted in a lower number of long and high molecular weight transcripts, respectively. The higher number of full-length functional transcripts per unit of RNA mass subsequently produced more proteins in cell-free translation assays. Although immunoblotting indicated that T7 urea transcripts do not necessarily contain less dsRNA than silica-purified T7 mRNA, much less interferon activity was noticed on RAW-Dual^TM^ reporter cells. Moreover, immunoblotting also indicated that the residual dsRNA after urea T7 IVT could further be cleared by an additional cellulose purification step [8]. Ultimately, urea supplementation combined with cellulose purification resulted in the lowest interferon responses thereby indicating mRNA of highest quality. An additional advantage of using urea is that it is an endogenous compound which is also a widely used component of FDA-approved medicines and cosmetic formulations [20]. It is therefore unlikely that urea will pose downstream health hazards if trace contaminants are still present.

In summary, whereas VSW3 RNAP failed to efficiently produce m1Ψ -modified mRNA, we provide evidence that supplementation of 1 M urea to T7 RNAP IVT reactions successfully reduced both shorter and longer transcript impurities. Urea supplementation resulted in a higher fraction of full-length transcripts after IVT, as well as in higher protein production yield, but still required an additional cellulose purification step to remove residual dsRNA. Considering the high influence of Mg^2+^ and NTP concentrations and their relative ratios on the IVT reaction [21], further investigation is warranted on how urea addition affects the reaction optimum of these essential components. In addition, it is unclear how urea supplementation will affect the base pairing process during co-transcriptional capping efficiency [22].

## Materials and methods

### In vitro transcription

We set out to produce therapeutically relevant mRNA and therefore chose to supplement our IVT reactions with 1-methylpseudouridine (m1Ψ) triphosphate instead of UTP. In addition, an excess of NTPs in the IVT reaction can lead to aberrant transcript formation such as dsRNA. We therefore chose to perform all our IVT reactions with weighted NTPs, i.e. the supplementation of each individual NTP was offset to its relative frequency in the firefly luciferase (fluc) encoding template sequence (30.6% A, 29.6% C, 25.5%G, 14.3% m1Ψ) whereby a value of 25% corresponds to 10 mM final concentration. For T7 RNAP IVT reactions (New England Biolabs #E2040S), we used a Cleancap AG-compatible promoter sequence (TAATACGACTCACTATAAGG) whereas VSW3 RNAP IVT reactions (16 hours at 25°C) utilized the VSW3 RNAP promoter sequence (TTAATTGGGCCACCTATA) with a G as first nucleotide of the transcript, as described by Xia et al. [13]. Additional T7 RNAP IVT reactions containing 1 M urea (final concentration) were performed in parallel and all IVT reactions were directly followed up by DNAse I digestion, and one or two silica column cleanups (NEB #T2040S). A Vaccinia capping enzyme reaction was used where indicated (Cellscript #C-SCCE0625). Spectrophotometer measurements were performed on a DeNovix DS-11 FX+ spectrophotometer/fluorometer.

### Cell-free translation assays

500 ng mRNA was dissolved in 13 µl nuclease-free (NF) water and combined with 10 µl nuclease-treated rabbit reticulocyte lysate (RRL, Promega #L4960) containing all amino acids. 21 µl of this mixture was then incubated for 120 minutes at 30°C and 1 µl D-luciferin substrate (0.75 mg/ml) was added to this reaction so that continuous luminescence readouts could be performed in a white 96-well plate on a Tecan Spark plate reader. The AUC of the curves was used to calculate relative protein production (%).

### Capillary gel electrophoresis

200 ng of each mRNA sample was loaded onto an Agilent Bioanalyzer 2100 system using the Agilent RNA 6000 Nano kit (#5067–1511) according to the manufacturer’s instructions but excluding the 2-minute heating at 70°C prior to loading (i.e. CGE was performed at non-denaturing conditions). Electropherogram smear analysis was performed on the Agilent 2100 Expert software (version B.02.08.SI648) using fixed regional borders as indicated in Figure 2.

### Asymmetric field flow fractionation

AF4 was conducted using a AF2000 Multiflow FFF (PN) coupled with a Multi-Angle Light Scattering instrument (PN3621 MALS Detector) and a UV Absorbance Detection System (Shimadzu SPD-20A/20AV) at 230 and 260 nm. The instrument included the necessary isocratic pump(s), degasser, autosampler injectors, and automatic fraction collector. The following conditions were used for the analyses: (i) sample injected volume, 2–28 µL of sample (total injected mass between 0.5 and 5 µg); (ii) membrane, 10 kDa regenerated cellulose; (iii) mobile phase, 50 mM TRIS-HCl +20 mM NaCl, pH 8; (iv) spacer, 350 µm; (v) injection flow, 0.2 mL.min^−1^; and (vi) detector flow, 0.5 mL.min^−1^; (vii) focus flow and time, 2 mL.min^−1^ for 6 min. Separation was done by [1] crow-flow constant at 0.6 mL.min^−1^ for 5 min [2], linear crossflow decay from 0.6 to 0.6 mL.min^−1^ for 50 min [4], flow constant at 0.1 mL.min^−1^ for 5 min and [7] flow constant at 0 mL.min^−1^ for 10 min.

### Immunoblotting

1–5 µg of mRNA was diluted in 200 ul RNase-free water and loaded onto pre-wetted nylon membrane (Whatman Nytran™ SuperCharge) using a vacuum system (Bio-Dot Microfiltration Apparatus 170–6545, BioRad). dsRNA ladder (NEB #N0363S) was serially diluted and loaded onto the same membrane as positive control. Next, the membrane was air-dried and blocked using 5% skim milk in TBS-T for 1 hour at room temperature. After three 5-minute washes in TBS-T, the membrane was incubated with anti-dsRNA IgM antibody (#RNT-SCI-10030005, Jena Bioscience) overnight at 4°C (1:7 dilution in 1% skim milk in TBS-T). After three 10-minute washes in TBS-T, the membrane was then incubated with a-IgM secondary antibody (ab5929, Abcam) for 1 hour at room temperature (1:500 dilution in 1% skim milk in TBS-T). After another three 10-minute washes in TBS-T, the membrane was incubated with ABC staining solution (Ultra-Sensitive ABC Peroxidase Standard Staining Kit, #32050 Thermo Scientific™) for 30 minutes at room temperature. The membrane was washed three times for 5 minutes in TBS-T, incubated for 1 minute with the ECL substrate (SuperSignal™ West Pico PLUS Chemiluminescent Substrate, #34580 Thermo Scientific™), and imaged (ChemiDoc MP, BIO RAD).

### Reporter cell assays

Fifty thousand RAW-Dual^TM^ cells (Invivogen #rawd-ismip) per well were cultured for 24 hours in a 96-well plate using 200 µl complete medium (RPMI 1640, 2 mM L-glutamine, 25 mM HEPES, 10% heat-inactivated foetal bovine serum, 100 U/ml Penicillin/Streptomycin). The next day, each well was transfected with 40 µl mRNA-lipofectamine Messenger Max (50 ng mRNA, 0.4 µl LFMM) in OptiMEM, according to the manufacturer’s instructions. After 24 hours, 20 µl supernatant of each well was transferred to a new 96-well plate and 50 µl Quanti-Luc substrate was added per well, immediately followed by luminescence readout on a Tecan Spark plate reader.

### Statistics

We performed one-way ANOVA. When statistically significant differences between the analysed groups were seen, we performed a post-hoc Tukey’s multiple comparisons test and depicted the p-values in the respective figures. Graphpad Prism v 10.1.0 (316) was used. *p < 0.05, **p < 0.01, ***p < 0.001, ****p < 0.0001, non-significant (ns, *p* > 0.05). and reported the uncorrected p-values. Every analysis we performed is depicted on the graph so that readers can perform any correction for multiple comparisons of choice.

## Supplementary Material

Supplemental Material

## Data Availability

The authors confirm that the data supporting the findings of this study are available within the article and its supplementary materials.
