# Supplementary figures and images for: Urea supplementation improves mRNA in vitro transcription by decreasing both shorter and longer RNA byproducts
Source: RNA Biol. 2024 Feb 27;21(1):1–6. doi: 10.1080/15476286.2024.2321764 (PMC10900265; doi:10.1080/15476286.2024.2321764)

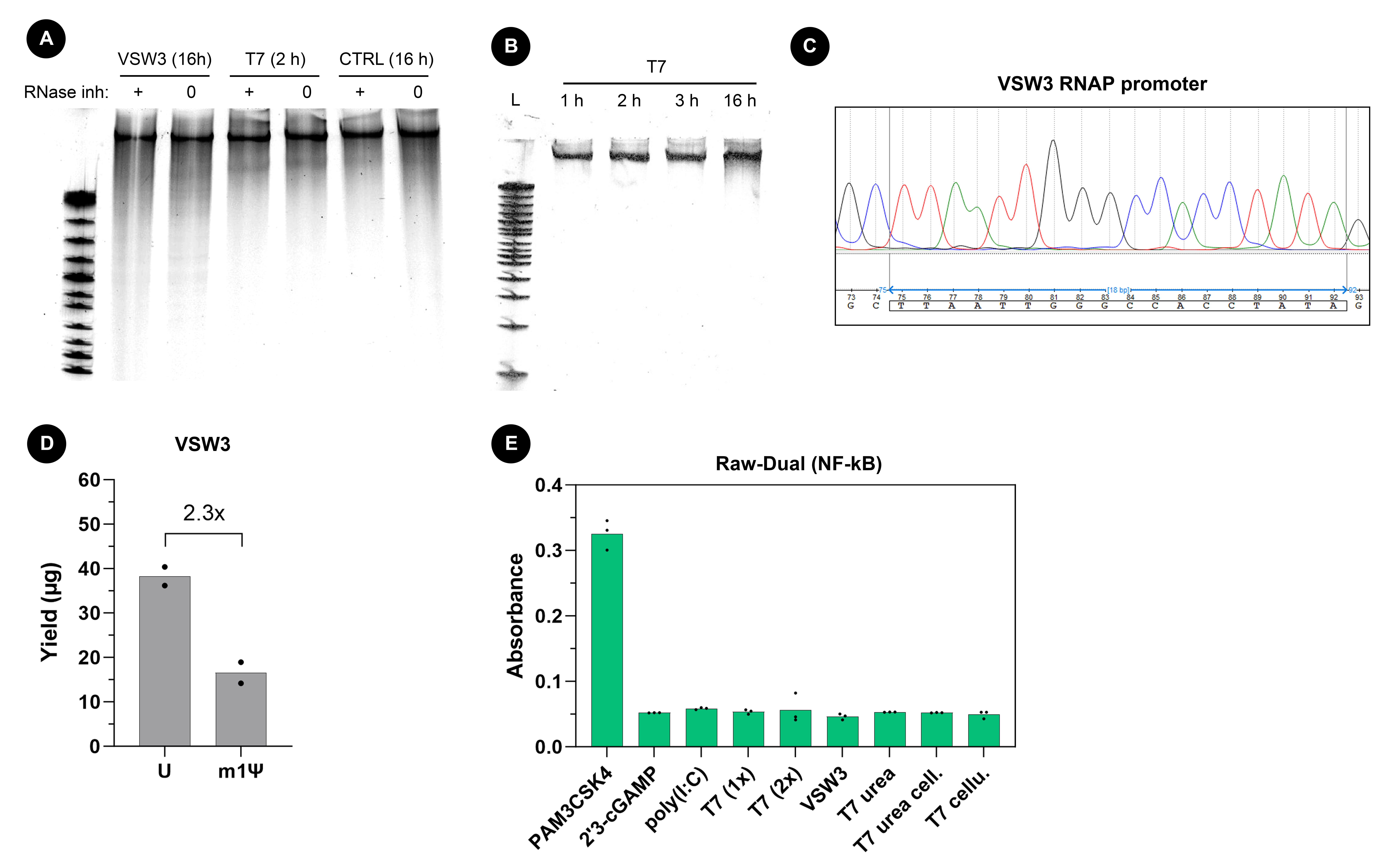

Supplement: Supplemental Material [file KRNB_A_2321764_SM0280.tif]
